# COVID-19 Pandemic and Trends in Clinical Trials: A Multi-Region and Global Perspective

**DOI:** 10.3389/fmed.2021.812370

**Published:** 2021-12-24

**Authors:** Satoshi Nishiwaki, Yuichi Ando

**Affiliations:** Department of Advanced Medicine, Nagoya University Hospital, Nagoya, Japan

**Keywords:** clinical trial, COVID-19 pandemic, non-COVID-19 activity, clinical development, new drug application

## Abstract

To evaluate the effect of the COVID-19 pandemic on clinical development, the number of newly started clinical trials in each geographical region between January 2018 and December 2020 were calculated based on data from the ClinicalTrials.gov database. Data regarding new drug applications were obtained from European Medicines Agency monthly reports, pharmaceutical company press releases, and the archives of the Drugs.com database. The mean percentage change in newly started clinical trials for diseases other than COVID-19 between each month in 2019 and the corresponding month in 2020 was −7.5%, with the maximum of −57.3% observed between April 2019 and April 2020. Similarly, the mean percentage change of reported results for each month in 2019 and 2020 was −5.1%, with the maximum of −27.4% observed in July 2020. The activity of clinical trials was decreased as the number of COVID-19 patients was increased, and a statistically negative correlation was observed between the prevalence of COVID-19 and the percentage decrease in the number of clinical trials stared or reported results. As for new drug submissions, decreases were observed in the latter half of 2020 compared with the same period during the previous year, for each indicator. A considerable decline in non-COVID-19 activity for all indicators regarding clinical developments was suggested during the first wave of the COVID-19 pandemic. It is important to recognize the situation and continue to make efforts to conduct clinical trials for both COVID-19 and no-COVID-19 for new medical developments in the future.

## Introduction

The coronavirus disease 2019 (COVID-19) pandemic is having an enormous impact on our various activities, with total case numbers reaching more than 240 million as of October 2021. Heath systems and economies around the world have been severely damaged; therefore, there is a concern that the development of medical technology, including the development of new drugs, will also be hindered. The U.S. Food and Drug Administration (FDA) has released guidance for conducting clinical trials during the COVID-19 pandemic (1). In addition to the flexible regulatory response under the pandemic, it is also important to balance the budget and manpower between non-COVID-19 and COVID-19 studies. Although the importance of maintaining the integrity of clinical trials has been pointed out (2), analyses of the impact of the COVID-19 pandemic on conducting clinical trials, especially during the first wave, have been inadequate (3).

Crucial clinical trials are being conducted internationally, which can lead to new drug applications in each participating country or region.

Although the reasons remain unclear, differences in the geographical distribution of COVID-19 and its severity have been reported (4). Therefore, when analyzing the impact of COVID-19 on clinical trials, it is necessary to consider regional differences in the prevalence of COVID-19. Furthermore, because the potential for conducting clinical trials varies from region to region, it is desirable to make comparison with past implementation status, and not just perform a cross-sectional snapshot of activity at one point in time. Therefore, in this study, we analyzed the impact of COVID-19 on clinical trials and subsequent new drug applications, based on the number of monthly clinical trials being conducted and the burden of COVID-19 in each region. While results such as the rapid development of COVID-19 vaccines have been achieved to overcome the COVID-19 pandemic (5–7), medical development for non-COVID-19 is also important. Therefore, this study mainly focused on clinical trials for non-COVID-19.

## Methods

### Number of Clinical Trials

The data relating to the number of clinical trials being conducted that were included in this study were obtained from the ClinicalTrials.gov database, operated by the U.S. National Library of Medicine (8). “Study type” was limited to “Interventional Studies (Clinical Trials).” Monthly data were extracted by specifying the date using “Study Start” in the advanced search. Regional data were extracted according to the “On Map” tab in the search results. The countries included in each region are listed in Supplementary Table 1. The number of clinical trials for diseases other than COVID-19 was calculated by subtracting the number of clinical trials for COVID-19 (entering “COVID-19 OR SARS-CoV-2” in the “Condition or disease” field) from the number of all clinical trials. Monthly data regarding reported results were extracted by specifying the date using “Results First Posted” in the advanced search. Among these, studies labeled with the status “Completed” were identified as completed clinical trials with results.

### Number of COVID-19 Cases

The total number of COVID-19 cases was obtained from the website “Worldometer Coronavirus” (9). The total number of COVID-19 cases at the end of the previous month was used to examine the impact on clinical research activities during that month, extracted from the cumulative graph of total cases by country.

### New Drug Applications

The number of product applications submitted to the European Medicines Agency (EMA) was calculated from their monthly statistics report (January 2018 to December 2020) (10). The number of new product applications submitted was based on the total of “New products” for “Non-orphan medicinal products” and “Orphan medicinal products.” To estimate the number of new applications to the FDA, we counted the number of pharmaceutical company press releases regarding a New Drug Application (NDA) or Biologics Licensing Application (BLA). We also estimated the activity regarding new drug applications using the Drugs.com databases (11). We analyzed the number of topics related to new drug applications (including BLA) published in the monthly New Drug Applications Archives on the Drugs.com.

### Statistical Analysis

The percentage change in the number of clinical trials was calculated by dividing the number of cases in each month by the number of cases during the same month of the previous year. A percentage change of >0% (+XX%) means there was an increase, while a percentage change of < 0% (-XX%) means there was a decrease. Pearson's correlation coefficient (12) was calculated and used to analyze correlation between the percentage change in clinical trials and the prevalence of COVID-19. The correlation was examined both at the peak of the decline in activities and at the monthly average for 2020. We determined that there was a positive correlation between the percentage change in clinical trials and the prevalence of COVID-19 when the correlation coefficient value 0.5 or more, and there was a negative correlation when the correlation coefficient was −0.5 or less (13, 14).

## Results

### Change in the Number of Newly Started Clinical Trials

**Figure 1:**
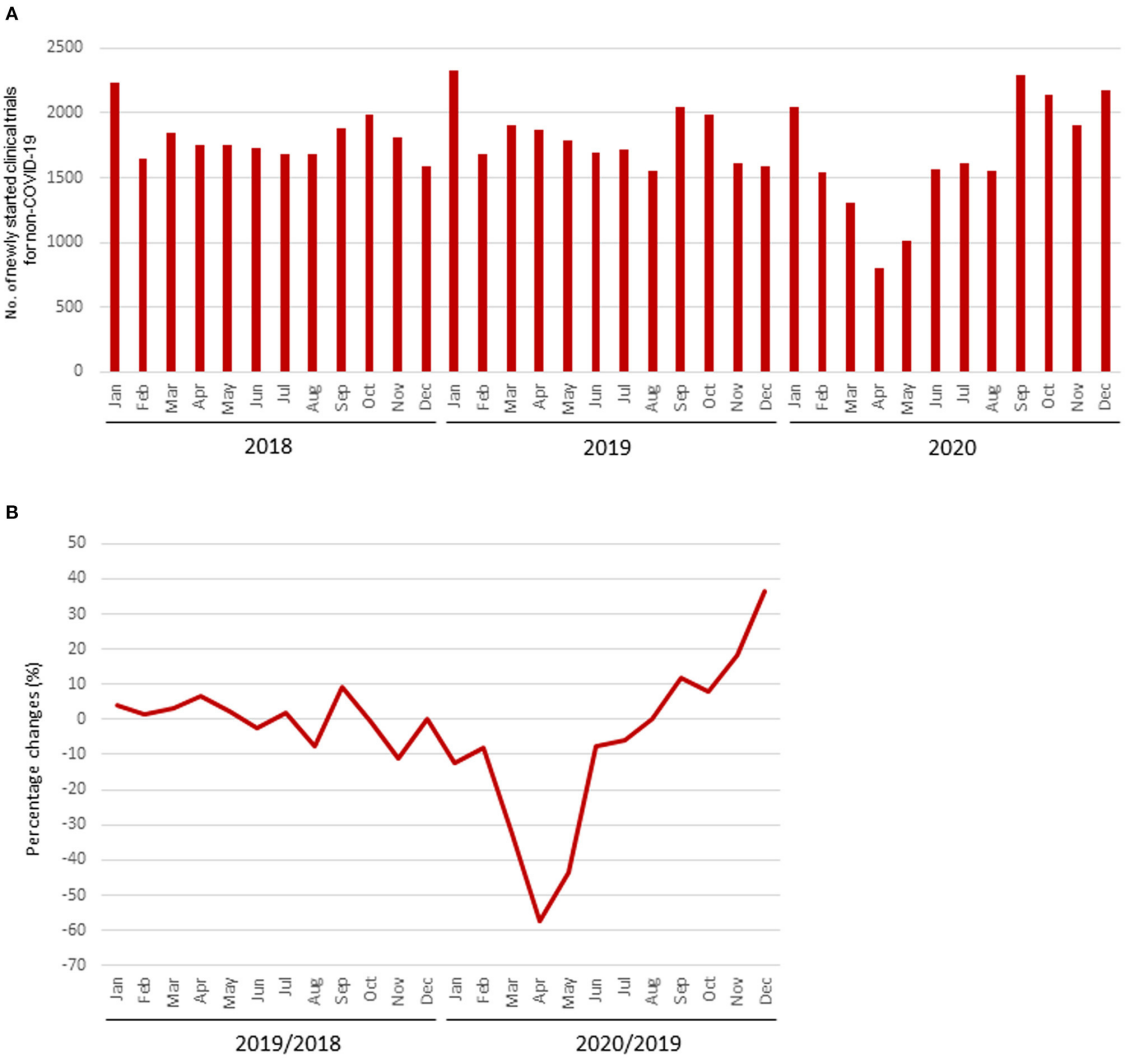
Number of newly started clinical trials. **(A)** Number of newly started clinical trials for diseases other than COVID-19; **(B)** Percentage change of newly started clinical trials for diseases other than COVID-19.

The mean numbers of clinical trials started each month during 2018, 2019, and 2020 were 1,802 [standard deviation (SD), 168], 1,813 (SD, 216), and 1,857 (SD, 394), respectively, while the number for diseases other than COVID-19 in 2020 was 1,663 (SD, 450). The mean percentage change for clinical trials for diseases other than COVID-19 in each month 2018 and 2019 was +0.5% (SD, 5.4%), whereas that in 2019 and 2020 was −7.5% (SD, 25.3%). The decrease from March to May 2020 was notable compared with the same period during the previous year, and the number of newly started clinical trials for diseases other than COVID-19 decreased in all regions (Table 1, Figure 1).

**Table 1:** Number of newly started clinical trials.

**Year**		**January**	**February**	**March**	**April**	**May**	**June**	**July**	**August**	**September**	**October**	**November**	**December**	**Total**
2018	All	2240	1652	1847	1751	1749	1735	1687	1683	1880	1992	1817	1591	21624
2019	All	2326	1677	1908	1868	1786	1691	1716	1552	2051	1982	1612	1593	21762
2020	All	2055	1588	1422	1226	1358	1823	1851	1737	2494	2312	2079	2345	22290
**2020**	**Other than COVID-19**													
	Global	2042	1544	1310	797	1012	1563	1617	1556	2295	2143	1908	2177	19964
	Percentage change* (%)	−12.2	−7.9	−31.3	−57.3	−43.3	−7.6	−5.8	+0.3	+11.9	+8.1	+18.4	+36.7	−8.3
	Africa	96	58	56	42	37	66	63	41	87	74	72	50	742
	%	−32.9	−37.0	−37.8	−53.3	−45.6	0.0	−32.3	−44.6	−6.5	−9.8	−8.9	−29.6	−28.7
	Central America	8	8	5	4	14	13	12	7	16	5	13	4	109
	%	−20.0	−33.3	−64.3	−75.0	+40.0	+8.3	−47.8	−12.5	+6.7	−64.3	0.0	−66.7	−31.4
	East Asia	250	154	216	183	247	302	296	270	297	268	244	255	2,982
	%	−25.4	−25.2	−27.0	−40.6	−17.7	+3.4	+2.4	+5.1	+23.8	+0.4	+1.2	−11.1	−10.1
	Japan	19	32	25	8	18	35	37	24	36	30	26	11	301
	%	−36.7	+33.3	−41.9	−78.9	−55.0	+9.4	+12.1	+71.4	+38.5	+11.1	−16.1	−66.7	−18.9
	Europe	549	446	320	142	226	436	379	283	599	457	409	360	4,606
	%	−16.7	−12.0	−40.3	−72.9	−55.1	−2.5	−6.0	−19.4	−1.5	−23.1	−7.7	−6.0	−22.7
	Middle East	95	89	71	30	54	63	62	63	69	59	62	68	785
	%	−40.6	−4.3	−33.6	−72.2	−47.1	−16.0	−42.1	−7.4	−22.5	−33.7	−38.6	−16.0	−33.5
	North America	822	653	443	229	306	520	642	685	782	824	698	816	7,420
	%	−4.1	−5.9	−42.0	−70.2	−59.7	−28.8	−16.0	−1.0	−6.9	−0.1	+8.9	+30.8	−17.2
	Canada	102	95	64	29	34	69	70	73	115	91	80	62	884
	%	−30.6	+1.1	−48.8	−74.3	−67.9	−37.8	−35.8	−24.7	−21.2	−2.2	−9.1	−37.4	−33.4
	Mexico	6	16	7	2	4	8	9	6	12	12	9	9	100
	%	−66.7	−23.8	−30.0	−88.9	−80.0	−61.9	−60.9	−76.0	−33.3	−40.0	−43.8	−25.0	−55.0
	United States	741	583	391	208	285	484	606	639	688	745	635	764	6,769
	%	−1.1	−6.7	−42.7	−70.2	−57.8	−25.8	−11.4	+5.3	−3.8	−1.5	+10.6	+36.7	−15.1
	North Asia	24	44	18	13	18	33	31	23	33	32	22	20	311
	%	−45.5	+100.0	−45.5	−71.1	−45.5	−26.7	+3.3	+9.5	−15.4	−11.1	−26.7	−31.0	−23.6
	Pacifica	34	35	38	18	23	53	44	44	53	50	56	29	477
	%	−17.1	+2.9	−25.5	−51.4	−41.0	+26.2	−17.0	+25.7	+12.8	+42.9	+69.7	−44.2	−4.4
	South America	39	35	33	20	23	36	31	32	33	34	31	28	375
	%	−40.9	−18.6	−37.7	−71.0	−46.5	−44.6	−32.6	−38.5	−49.2	−22.7	−26.2	−28.2	−40.2
	South Asia	47	34	19	7	13	30	26	30	30	18	23	16	293
	%	−14.5	+30.8	−47.2	−70.8	−50.0	+20.0	−18.8	+30.4	−21.1	−43.8	−23.3	−64.4	−25.3
	Southeast Asia	40	23	20	13	23	33	33	33	42	46	31	25	362
	%	−24.5	−41.0	−50.0	−60.6	−52.1	−25.0	−8.3	−28.3	−17.6	+21.1	−6.1	−26.5	−26.9

* *Percentage change compared the number of clinical trials for other than COVID-19 in 2020 with those in the same month of 2019*.

**Figure 2:**
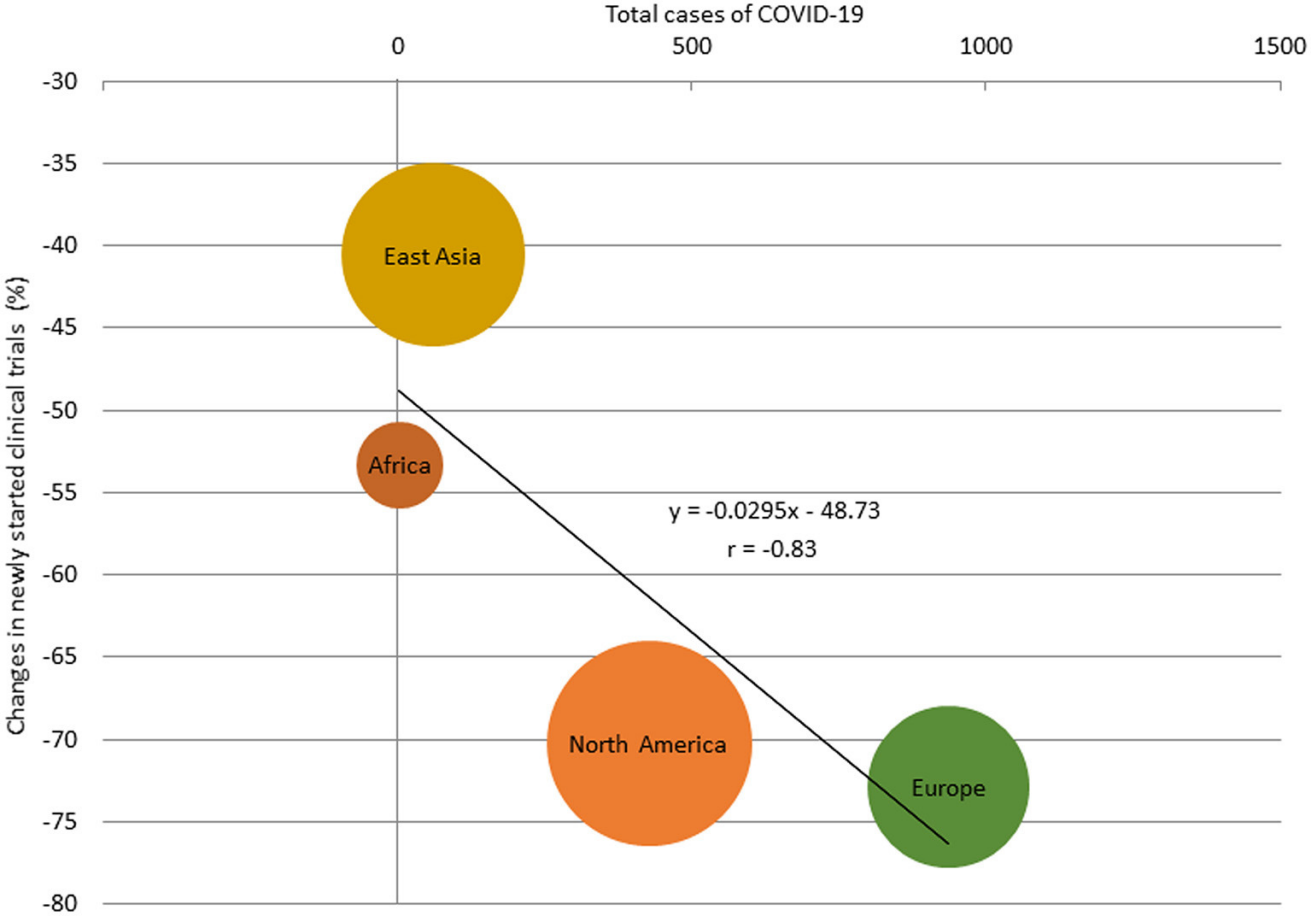
Relationship between total cases of COVID-19 and percentage change of the number of newly started clinical trials for diseases other than COVID-19 in regions with more than 30 studies, April 2020. The size of the bubbles reflected the number of studies.

The percentage change in newly started clinical trials for diseases other than COVID-19 in April 2020 by region are shown in Supplementary Figure 1, and the relationship between the percentage decrease and the prevalence of COVID-19 by region is shown in Supplementary Figure 2. There was a correlation between the percentage change and the prevalence of COVID-19 in regions where more than 30 clinical trials were started in April 2020 (*r* = −0.83; Figure 2). The percentage change in newly started clinical trials for diseases other than COVID-19 in April 2020 and the total cases of COVID-19 per 1 million population in the end of March 2020 in North America, Europe, East Asia, and Africa were −70.2 and 428, −72.9% and 937, −40.6% and 61, and −53.3% and 4, respectively. There was also negative correlation between the average monthly number of clinical trials and COVID-19 infections in 2020 (*r* = −0.56; Supplementary Figure 3).

The number of clinical trials for diseases other than COVID-19 that were started during the fourth quarter of 2020 showed signs of recovery, but this was due to the increase in the number started in the United States. In December 2020, the number of clinical trials started for diseases other than COVID-19 was still less than that in during the same month of the previous year in regions other than the United States. On the other hand, the total number of clinical trials (COVID-19 and non-COVID-19) recovered rapidly and has exceeded the number in the same month of the previous year since June 2020.

### Change in the Number of Clinical Trial Results Reported

**Figure 3:**
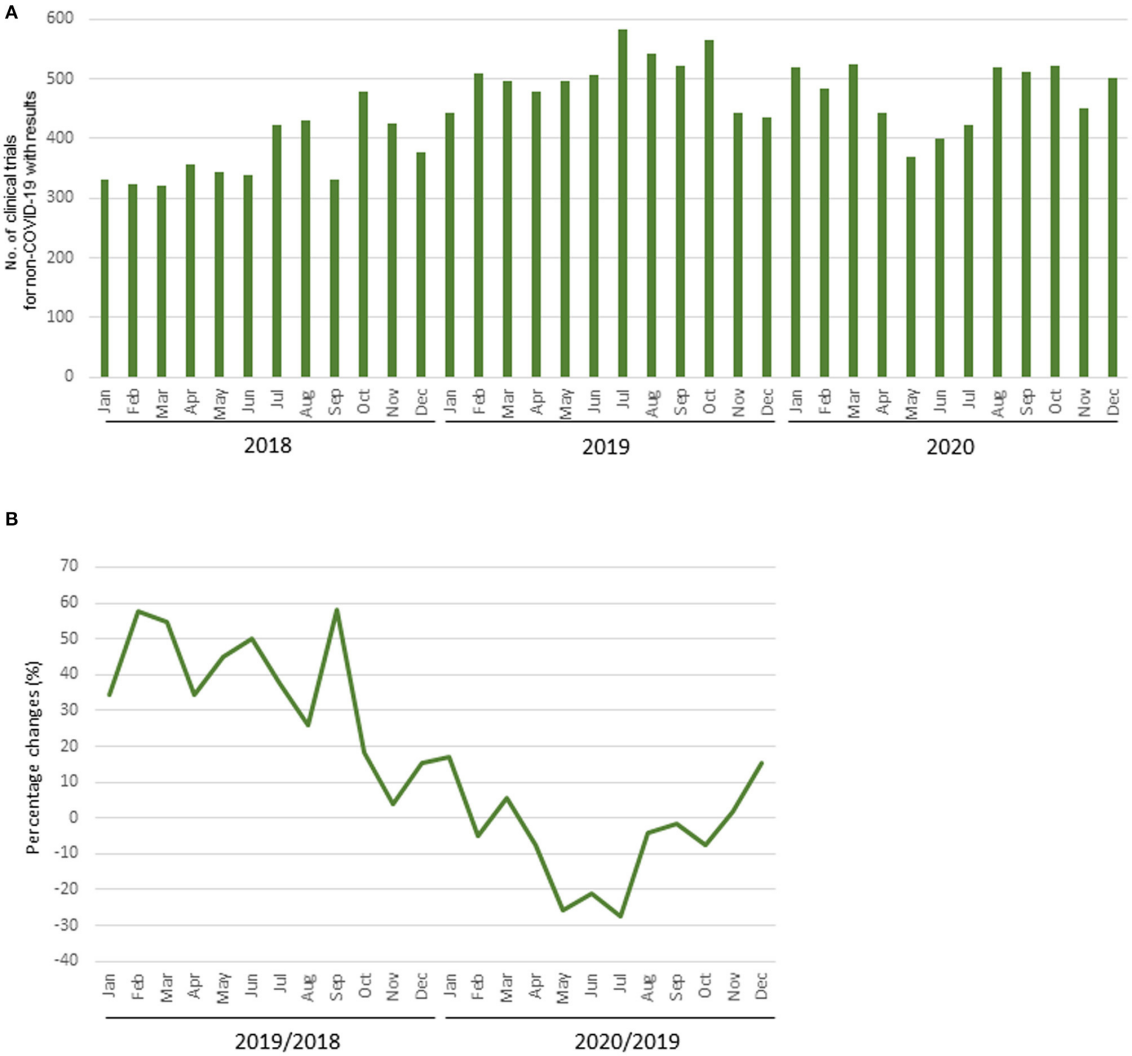
Number of clinical trial results reported. **(A)** Number of clinical trials for diseases other than COVID-19 with results; **(B)** Percentage change of clinical trials for diseases other than COVID-19 with results.

The mean numbers of clinical trial results reported each month in 2018, 2019, and 2020 were 373 (SD, 50), 502 (SD, 45), and 474 (SD, 52), respectively, and the number for diseases other than COVID-19 in 2020 was 472 (SD, 51). The mean percentage change in clinical trials for diseases other than COVID-19 each month during 2018 and 2019 was +36.3% (SD, 17.0%), whereas that during 2019 and 2020 was −5.1% (SD, 13.7%). The decrease from May to July 2020 was notable compared with the same period during the previous year (Table 2, Figure 3).

**Table 2:** Number of clinical trials with results.

**Year**		**January**	**February**	**March**	**April**	**May**	**June**	**July**	**August**	**September**	**October**	**November**	**December**	**Total**
2018	All	330	323	322	357	343	339	423	431	330	478	425	378	4479
2019	All	444	510	498	480	497	508	583	543	522	565	442	436	6028
2020	All	520	484	525	444	369	401	424	524	515	523	454	505	5688
**2020**	**Other than COVID-19**													
	Global	520	484	525	444	369	401	423	520	513	522	450	502	5,673
	Percentage change* (%)	+17.1	−5.1	+5.4	−7.5	−25.8	−21.1	−27.4	−4.2	−1.7	−7.6	+1.8	+15.1	−5.9
	Africa	14	13	13	7	4	8	14	8	8	11	17	14	131
	%	−26.3	−13.3	−13.3	−50.0	−66.7	−27.3	−26.3	−50.0	−55.6	−54.2	−19.0	+55.6	−32.1
	Central America	8	9	12	8	12	15	14	4	11	11	6	9	119
	%	0.0	+50.0	+9.1	0.0	+140.0	+200.0	+100.0	−63.6	+57.1	−26.7	−57.1	+80.0	+16.7
	East Asia	49	55	49	35	38	43	55	51	32	45	48	63	563
	%	−12.5	−1.8	−12.5	−5.4	+5.6	−17.3	−15.4	−10.5	−50.8	−19.6	+41.2	+43.2	−8.3
	Japan	22	24	24	9	15	16	22	23	16	23	21	26	241
	%	−24.1	−20.0	−25.0	−47.1	0.0	−40.7	−12.0	−4.2	−44.8	−8.0	+5.0	+4.0	−19.1
	Europe	115	112	110	102	79	98	101	107	90	103	93	95	1,205
	%	−12.2	−1.8	−6.0	−9.7	−14.1	−26.9	−22.3	−12.3	−28.6	−29.9	−19.1	−10.4	−16.7
	Middle East	27	26	20	24	21	21	23	17	18	27	26	22	272
	%	+3.8	+100.0	−23.1	+50.0	−4.5	+5.0	+27.8	−34.6	+5.9	−15.6	+18.2	+10.0	+5.4
	North America	407	382	411	344	287	312	333	404	410	431	354	394	4,469
	%	+31.7	+22.0	+18.4	−8.5	−27.7	−13.1	−13.3	+5.8	+13.3	+7.7	+13.5	+17.6	+4.5
	Canada	53	45	42	55	56	56	46	42	43	53	38	50	579
	%	+15.2	−4.3	−22.2	+10.0	+9.8	−3.4	+2.2	−16.0	−20.4	−5.4	−13.6	+2.0	−4.1
	Mexico	13	17	16	9	12	15	11	12	12	22	14	13	166
	%	−13.3	+70.0	+14.3	−43.8	−7.7	+50.0	−35.3	0.0	+9.1	+15.8	−12.5	−38.1	−4.6
	United States	393	375	399	318	268	300	323	396	399	408	344	383	4,306
	%	+33.2	+26.3	+19.8	−13.1	−30.6	−12.3	−10.5	+8.8	+19.8	+6.8	+17.0	+22.4	+5.9
	North Asia	28	25	23	21	15	27	28	23	17	26	38	31	302
	%	+40.0	−7.4	−20.7	−32.3	−28.6	−18.2	−12.5	−32.4	−32.0	−13.3	+58.3	+40.9	−7.9
	Pacifica	30	26	23	28	37	29	30	24	26	33	27	20	333
	%	−21.1	+8.3	−28.1	−12.5	+37.0	−14.7	+7.1	−4.0	+52.9	0.0	+17.4	−13.0	−0.9
	South America	25	24	22	16	15	16	25	15	20	21	21	22	242
	%	+8.7	+26.3	+10.0	−38.5	−21.1	−23.8	−10.7	+36.4	0.0	−32.3	+61.5	+15.8	−3.2
	South Asia	7	10	11	4	6	5	9	2	12	8	6	9	89
	%	0.0	0.0	+57.1	−50.0	−14.3	−50.0	−10.0	−80.0	+71.4	0.0	+20.0	−10.0	−10.1
	Southeast Asia	12	13	13	18	13	21	18	5	8	15	13	15	164
	%	−36.8	−18.8	−35.0	+50.0	+8.3	+23.5	−14.3	−77.3	−11.1	0.0	0.0	−11.8	−15.0

* *Percentage change compared the number of clinical trials for other than COVID-19 in 2020 with those in the same month of 2019*.

**Figure 4:**
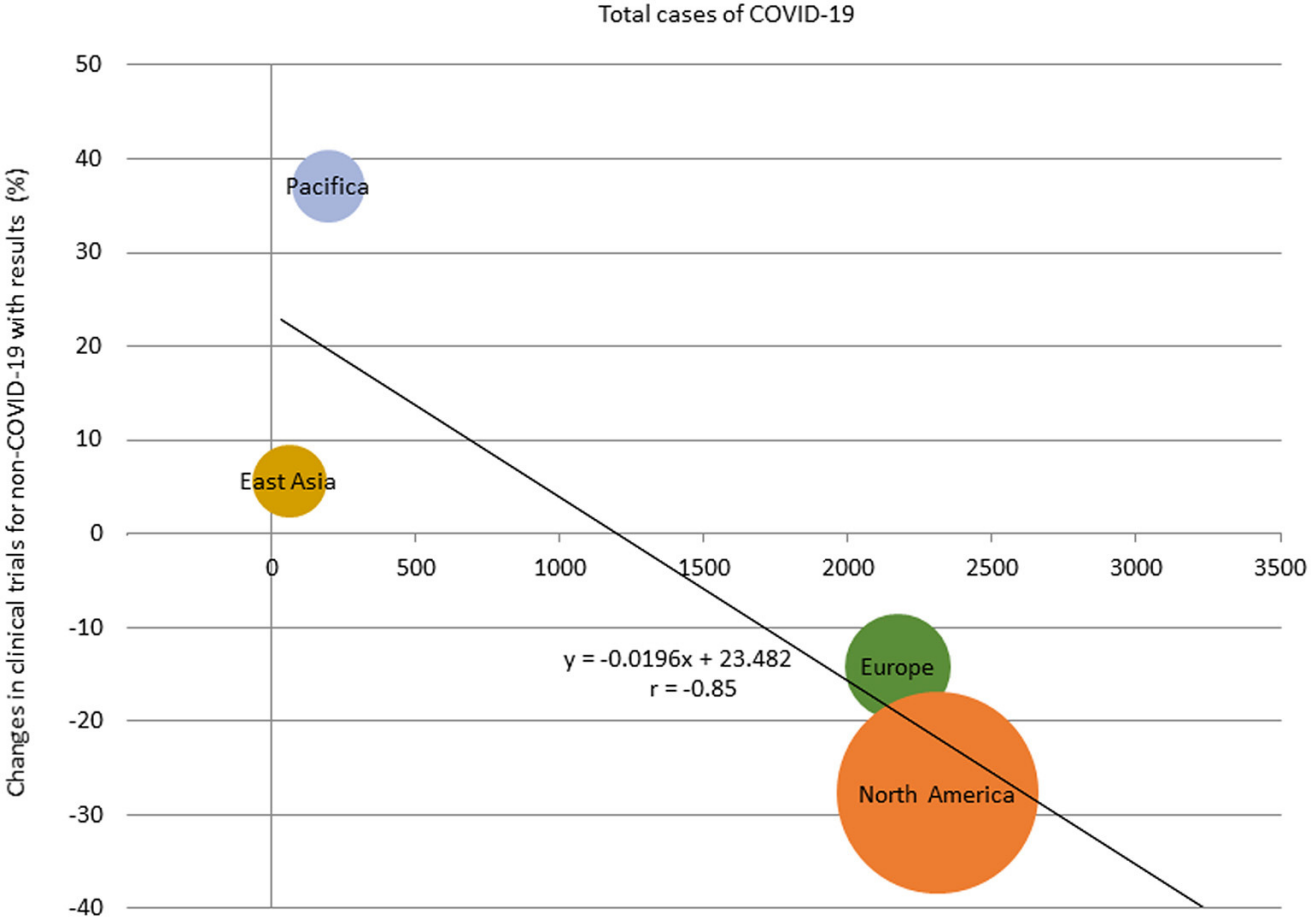
Relationship between total cases of COVID-19 and percentage change of the number of clinical trials with results in regions with more than 30 studies, May 2020. The size of the bubbles reflected the number of studies.

The percentage change in clinical trial results for diseases other than COVID-19 for May 2020 by region and the relationship between the percentage change and the prevalence of COVID-19 by region is shown in Supplementary Figures 4, 5. There was a correlation between the percentage change and the prevalence of COVID-19 in regions where the results of more than 30 clinical trial were posted in May 2020 (*r* = −0.85; Figure 4). The percentage change in clinical trial results for diseases other than COVID-19 in May 2020 and the total number cases of COVID-19 per 1 million population at the end of April 2020 in North America, Europe, East Asia, and Pacifica were −27.7% and 2,312, −14.1% and 2,172, +5.6% and 62, and +37.0% and 198, respectively. There was also negative correlation between the average monthly number of clinical trials and COVID-19 infections in 2020 (*r* = −0.61; Supplementary Figure 6).

A similar tendency was observed in the number of completed clinical trials with results in 2020. The mean number of completed clinical trials with results for diseases other than COVID-19 in 2020 was 373 (SD, 38). The mean percentage change for each month between 2019 and 2020 was −7.9% (SD, 12.5%). The decrease from May to July 2020 was notable compared with the same period during the previous year (Supplementary Table 2, Supplementary Figure 7). The total numbers of completed clinical trials with results in 2020 decreased compared with the number in 2019 in Africa, East Asia, Japan, Europe, Canada, North Asia, Pacifica, South Asia, and Southeast Asia.

### Change in the Number of Applications Submitted to Regulatory Authorities

**Figure 5:**
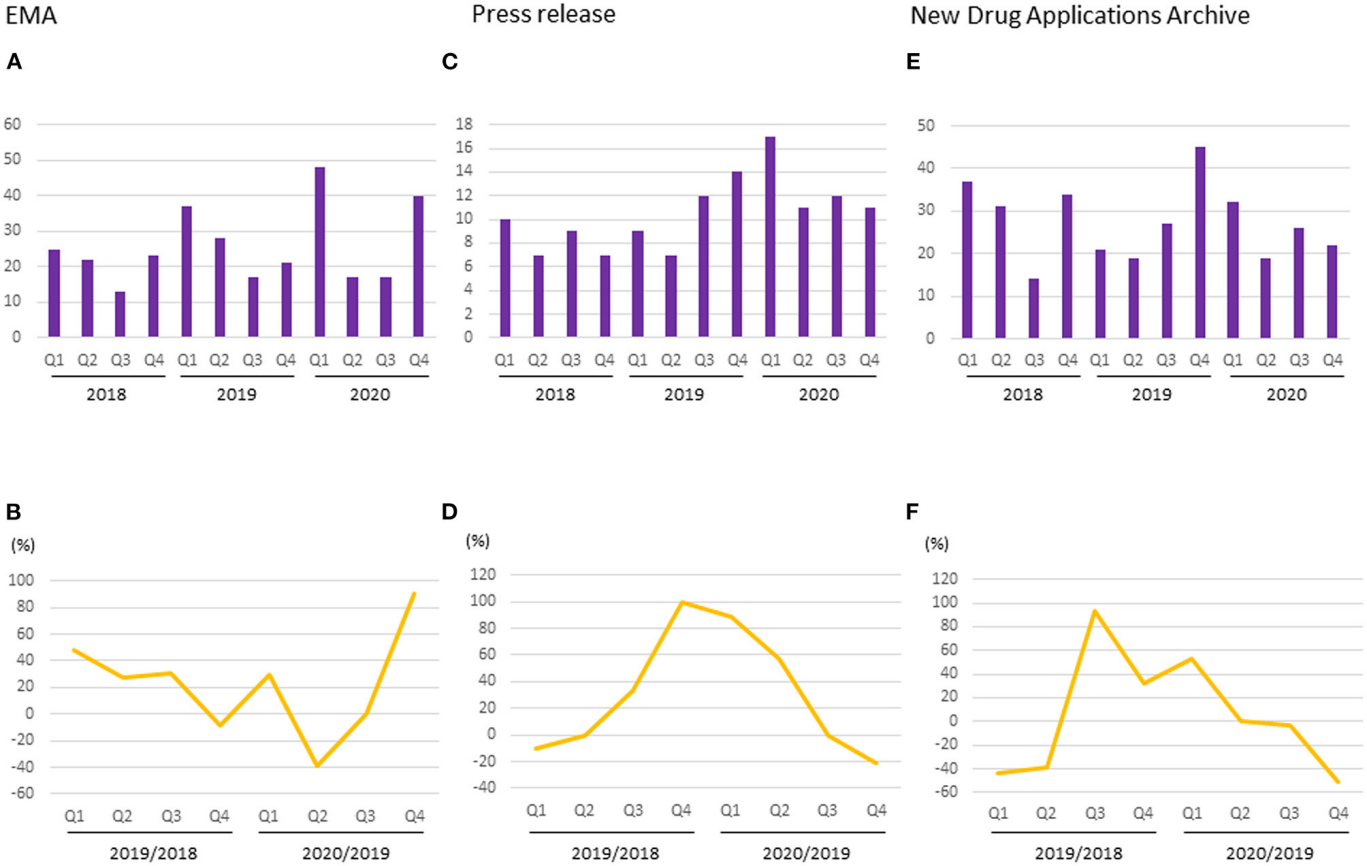
Number and percentage change of new drug applications to regulatory authorities. **(A)** Number of applications started in EMA; **(B)** Percentage change of applications started in EMA; **(C)** Number of pharmaceutical company press release regarding NDA or BLA to FDA; **(D)** Percentage change of pharmaceutical company press release regarding NDA or BLA to FDA; **(E)** Number of new drug application archives in the Drugs.com database; **(F)** Percentage change of new drug application archives in the Drugs.com database.

The median numbers of applications for new products submitted to the EMA per quarter in 2018, 2019, and 2020 were 11.5 (range 7–18), 12.5 (9–26), and 14.5 (7–29), respectively. The median percentage change for each quarter between 2018 and 2019 was ±24.3% (range 0.0–±44.4%), whereas that between 2019 and 2020 was ±1.6% (range −22.2–±38.5%). There was a decrease in applications during the second and third quarters of 2020 compared with the same quarters of the previous year (Figures 5A,B).

The median numbers of pharmaceutical company press releases regarding NDAs or BLAs per quarter in 2018, 2019, and 2020 were 8 (range 7–10), 10.5 (7–14), and 11.5 (11-17), respectively. The median percentage change for each quarter between 2018 and 2019 was +16.7% (range −10–+100%), whereas that between 2019 and 2020 was +28.6% (−21.4–+88.9%). There was a decrease in the number of press release during the fourth quarter of 2020 compared with the same quarter of the previous year (Figures 5C,D).

The median numbers of reports regarding submissions of new drug applications per quarter in the New Drug Applications Archives during 2018, 2019, and 2020 were 32.5 (range 14–37), 24 (19–45), and 24 (19–32), respectively. The median percentage change for each quarter between 2018 and 2019 was −3.2% (range −43.2–+92.9%), whereas between 2019 and 2020 it was −1.9% (−51.1–+52.4%). There was a decrease in submissions between the second and fourth quarters of 2020 compared with the same quarters of the previous year (Figures 5E,F).

## Discussion

Since the first case of COVID-19 was identified in Wuhan, China, in December 2019, the disease has spread rapidly all over the world (15). Between January and April 2020, lockdowns began in many countries or regions, which had a huge impact not only on medical care but also on economics (16). Although clinical trials are an essential process for the development of new medical treatments, they require a lot of time and are costly (17). Due to hospitals being overwhelmed with COVID-19 patients, the enrollment of new participants in clinical trials, as well as in-person visits of already registered participants, was prevented. In addition, the worsening financial situations of trial sponsors led to concern that fewer new clinical trials would be launched, and the number of incomplete trials would increase. Our results suggested that the first wave of the COVID-19 pandemic had a negative impact on clinical trials and subsequent drug development. Considering that it takes about 9 years to move from an Investigational New Drug Application to final FDA approval (18), this decrease in current clinical trials will affect new treatments for at least several years to come.

It is important to note that this study showed regional trends in addition to global trends of clinical trials conducted during the COVID-19 pandemic. Interestingly, there was a negative correlation between the number of COVID-19 cases and clinical trial activity. Demographic effects have been reported (19, 20), and it is clear that the number of COVID-19 cases varies by region. In East Asia, where the number of COVID-19 cases per 1 million population was <100 at the end of April 2020, the percentage decrease in the numbers of newly started clinical trials and reported results was modest compared with the decrease seen in Europe and the United States, where the number of COVID-19 cases per 1 million population was more than 2000. Given that international clinical trials are now frequently performed, if a region is unable to participate in a pivotal international clinical trial due to the COVID-19 epidemic, this will cause a delay in new drug development in that region. This could exacerbate the existing global inequalities. It is suggested that the control of COVID-19 by each country/region is also important for the development of future treatments in that country/region.

By the end of 2020, the numbers of newly started clinical trials and reported results had recovered despite the ongoing COVID-19 pandemic, which might reflect the recovery of researchers and companies from the psychological impact of the first wave. However, the situation is not optimistic. In addition to the second and third waves of COVID-19 infection, more easily transmissible variants of SARS-CoV-2, the virus that causes COVID-19, have been reported (21). More explosive outbreaks of infection than in currently seen could limit both the medical profession and society to unprecedented levels, as happened during the first wave of COVID-19. In such a scenario, it is necessary to consider a system that distributes resources appropriately and equitably and continues as much as possible to conduct clinical trials for diseases other than COVID-19 for the future development of new treatments (2). The introduction of remote monitoring using information and communication technology (ICT) is one approach that can be implemented even during the COVID-19 pandemic. Flexible responses by regulatory authorities and/or journals to delays, interruption, and missing data in clinical trials may also be helpful for making the most effective use of existing data (22). On the other hand, there are also positive outcomes of the pandemic. Vaccines have been developed at an unprecedented rate (5–7). The cooperation of various stakeholders, including, patients, healthcare professionals, pharmaceutical companies, and regulatory authorities, has led to the rapid completion of clinical trials and approval of vaccines. In addition, several clinical trials have been performed at an incredible speed, such as trials of corticosteroids (23) and interleukin-6 antagonists (24), therapeutic drugs that have been approved for COVID-19 (25, 26). It is unlikely that COVID-19 will disappear in the near future, and therefore some medical resources will continue to be dedicated to COVID-19 even in the post-COVID era. It is necessary to consider budget and staffing levels, with a view to developing new treatments for diseases other than COVID-19.

To evaluate the impact of the COVID-19 pandemic on overall clinical development, we examined the number of newly started clinical trials, reported results, and new drug applications. Interestingly, all indicators showed a marked drop in quarters 2 and 3 of 2020 compared with the same period during the previous year, indicating the magnitude of the impact of the first wave of COVID-19 on clinical development. The decrease in the number of clinical trials started might reflect the increase in workload of medical staff and/or the financial situation of sponsors. The decrease in the number of completed studies might reflect the effect of delayed patient recruitment and/or required visits, and the decrease in the number of submitted applications might reflect the termination of studies before they are competed and/or the increased workload of the applicant company and/or the regulatory authority. Many experts are involved from the initiation of clinical trials to the submission of new drug applications, thus it can be seen that the COVID-19 pandemic affected various stages of clinical development.

There are several limitations to this study. First, we did not directly evaluate the effects of COVID-19 by following the progress of individual studies but analyzed trends based on publicly accessible data. Second, the clinical trials data were obtained from ClinicalTrials.gov. We did not include studies registered in other registries, such as the EU Clinical Trials Register (EuroCT) in the European Union and the Japan Primary Registries Network (JPRN) in Japan. We determined that using the data from ClinicalTrials.gov was the best way to understand the status of clinical trials worldwide. In addition, we selected this database because important trials are being conducted with FDA approval in mind and the FDA requires registration to the ClinicalTrials.gov database (27), although registration of trials conducted in non-Western countries might be inadequate. It should also be noted that the results posted in the ClinicalTrials.gov database were inadequate (28) and that the number was an approximation rather than the actual number of results reported. By comparing data with the same month of the previous year, we could clearly show the impact of COVID-19 on clinical trials worldwide. Third, only limited data were available on the number of submitted new drug applications. We could not find official data from the FDA or the Pharmaceuticals and Medical Devices Agency (PMDA) regarding the number of new applications for each submission period (29). However, because information about an approved drug and its approval date is made publicly available, it will be possible to evaluate the number of new approvals in another year or so.

During the first wave of the COVID-19 pandemic, there was a significant decline in clinical trial activity and subsequent new drug applications. This decline will affect the development of new treatments in the future, and it is necessary to recognize this situation and continue to make efforts to perform clinical trials for diseases other than COVID-19, as well as for COVID-19. Utilizing the COVID-19 prediction (30), it will be possible to reduce the impact by allocating resources so that activities for non-COVID clinical trials will not decrease before the rapid increase of COVID-19 infections in the future. It takes a lot of time and effort to launch and complete a clinical trial and to establish a new treatment, therefore a long-term perspective is required. Even in a situation in which the COVID-19 pandemic is not yet under control, it is important to implement concrete strategies that minimize the impact on new or ongoing clinical trials, based on our analysis of the first wave of the COVID-19 pandemic.

## Data Availability Statement

The original contributions presented in the study are included in the article/Supplementary Material, further inquiries can be directed to the corresponding author/s.

## Author Contributions

SN designed the research, performed the analysis and interpreted the data, and wrote the manuscript. YA supervised the analysis, interpreted the data, and revised the manuscript. All authors reviewed and approved the final draft.

## Funding

This work was partly supported by JSPS KAKENHI Grant No. JP 20K08730 and AMED under Grant No. JP21lk1503005.

## Conflict of Interest

YA reports grants and personal fees from Chugai Pharmaceutical Co., Ltd., Kyowa Hakko Kirin Co., Ltd., Nippon Kayaku Co., Ltd., Yakult Honsha Co., Ltd., Eli Lilly Japan K.K., Mochida Pharmaceutical Co., Ltd., Ono Pharmaceutical Co., Ltd., and Taiho Pharmaceutical Co., Ltd., personal fees from Novartis Pharma K.K., Merck Serono Co., Ltd., Bayer Holding Ltd., Bristol-Myers Squibb, Otsuka Holdings Co., Ltd., and Sawai Pharmaceutical Co., Ltd., grants and personal fees from Daiichi Sankyo Company, Ltd., grants from Eisai Co., Ltd., Hisamitsu Pharmaceutical Co., Inc., and Takeda Pharmaceutical Co., Ltd., personal fees from Tsumura & Co., Shionogi & Co., Ltd., Janssen Pharmaceutical K.K., and Pharma International Inc., outside the submitted work. The remaining author declares that the research was conducted in the absence of any commercial or financial relationships that could be construed as a potential conflict of interest.

## Publisher's Note

All claims expressed in this article are solely those of the authors and do not necessarily represent those of their affiliated organizations, or those of the publisher, the editors and the reviewers. Any product that may be evaluated in this article, or claim that may be made by its manufacturer, is not guaranteed or endorsed by the publisher.
