# Supplementary material for: COVID-19 Pandemic and Trends in Clinical Trials: A Multi-Region and Global Perspective
Source: Front Med (Lausanne). 2021 Dec 24;8:812370. doi: 10.3389/fmed.2021.812370 (PMC8739772; doi:10.3389/fmed.2021.812370)
Supplement: Supplementary file 1 [file Data_Sheet_1.docx]

**COVID-19 Pandemic and Trends in Clinical Trials: a Multi-region and Global Perspective**

*Satoshi Nishiwaki, Yuichi Ando*

**Supplementary Table 1** Region classification and included countries/regions

| Region | Country/region |
| --- | --- |
| Africa | Algeria |
|  | Angola |
|  | Benin |
|  | Botswana |
|  | Burkina Faso |
|  | Burundi |
|  | Cameroon |
|  | Central African Republic |
|  | Chad |
|  | Congo |
|  | Congo, The Democratic Republic of the |
|  | Côte D'Ivoire |
|  | Djibouti |
|  | Egypt |
|  | Equatorial Guinea |
|  | Eritrea |
|  | Ethiopia |
|  | Gabon |
|  | Gambia |
|  | Ghana |
|  | Guinea |
|  | Guinea-Bissau |
|  | Kenya |
|  | Lesotho |
|  | Liberia |
|  | Libyan Arab Jamahiriya |
|  | Madagascar |
|  | Malawi |
|  | Mali |
|  | Mauritania |
|  | Morocco |
|  | Mozambique |
|  | Namibia |
|  | Niger |
|  | Nigeria |
|  | Rwanda |
|  | Senegal |
|  | Sierra Leone |
|  | Somalia |
|  | South Africa |
|  | Sudan |
|  | Swaziland |
|  | Tanzania |
|  | Togo |
|  | Tunisia |
|  | Uganda |
|  | Zambia |
|  | Zimbabwe |
| Central America | Bahamas |
|  | Belize |
|  | Costa Rica |
|  | Cuba |
|  | Dominican Republic |
|  | El Salvador |
|  | Guatemala |
|  | Haiti |
|  | Honduras |
|  | Jamaica |
|  | Nicaragua |
|  | Panama |
|  | Puerto Rico |
|  | Trinidad and Tobago |
| East Asia | China |
|  | Hong Kong |
|  | Korea, Democratic People's Republic of |
|  | Korea, Republic of |
|  | Mongolia |
|  | Taiwan |
| Japan | Japan |
| Europe | Albania |
|  | Austria |
|  | Belgium |
|  | Bosnia and Herzegovina |
|  | Bulgaria |
|  | Croatia |
|  | Czech Republic |
|  | Denmark |
|  | Estonia |
|  | Finland |
|  | France |
|  | Germany |
|  | Greece |
|  | Hungary |
|  | Iceland |
|  | Ireland |
|  | Italy |
|  | Latvia |
|  | Lithuania |
|  | Luxembourg |
|  | Macedonia, The Former Yugoslav Republic of |
|  | Montenegro |
|  | Netherlands |
|  | Norway |
|  | Poland |
|  | Portugal |
|  | Romania |
|  | Serbia |
|  | Slovakia |
|  | Slovenia |
|  | Spain |
|  | Sweden |
|  | Switzerland |
|  | United Kingdom |
| Middle East | Cyprus |
|  | Iran, Islamic Republic of |
|  | Iraq |
|  | Israel |
|  | Jordan |
|  | Kuwait |
|  | Lebanon |
|  | Oman |
|  | Qatar |
|  | Saudi Arabia |
|  | Syrian Arab Republic |
|  | Turkey |
|  | United Arab Emirates |
|  | Yemen |
| North America | Canada |
|  | Mexico |
|  | United States |
| North Asia | Armenia |
|  | Azerbaijan |
|  | Belarus |
|  | Georgia |
|  | Kazakhstan |
|  | Kyrgyzstan |
|  | Moldova, Republic of |
|  | Russian Federation |
|  | Tajikistan |
|  | Ukraine |
|  | Uzbekistan |
| Pacifica | Australia |
|  | Australian Capital Territory |
|  | New South Wales |
|  | Northern Territory |
|  | Queensland |
|  | South Australia |
|  | Tasmania |
|  | Victoria |
|  | Western Australia |
|  | Fiji |
|  | New Caledonia |
|  | New Zealand |
|  | Papua New Guinea |
|  | Solomon Islands |
|  | Vanuatu |
| South America | Argentina |
|  | Bolivia |
|  | Brazil |
|  | Chile |
|  | Colombia |
|  | Ecuador |
|  | French Guiana |
|  | Guyana |
|  | Paraguay |
|  | Peru |
|  | Suriname |
|  | Uruguay |
|  | Venezuela |
| South Asia | Afghanistan |
|  | Bangladesh |
|  | Bhutan |
|  | India |
|  | Nepal |
|  | Pakistan |
|  | Sri Lanka |
| Southeast Asia | Brunei Darussalam |
|  | Cambodia |
|  | Indonesia |
|  | Lao People's Democratic Republic |
|  | Malaysia |
|  | Myanmar |
|  | Philippines |
|  | Singapore |
|  | Thailand |
|  | Vietnam |

**Supplementary Table 2** Number of completed clinical trials with results

| Year |  | January | February | March | April | May | June | July | August | September | October | November | December | Total |
| --- | --- | --- | --- | --- | --- | --- | --- | --- | --- | --- | --- | --- | --- | --- |
| 2020 | All | 403 | 377 | 411 | 360 | 306 | 319 | 329 | 431 | 406 | 395 | 358 | 397 | 4492 |
|  | Other than COVID-19 |  |  |  |  |  |  |  |  |  |  |  |  |  |
|  | Global | 403 | 377 | 411 | 360 | 306 | 319 | 328 | 427 | 404 | 394 | 355 | 395 | 4479 |
|  | Percentage change* (%) | +9.8 | -10.7 | +0.7 | -5.0 | -20.7 | -22.9 | -32.8 | -2.3 | -6.3 | -14.3 | +0.9 | +9.4 | -8.7 |
|  | Africa | 11 | 12 | 11 | 7 | 3 | 7 | 11 | 7 | 8 | 9 | 11 | 12 | 109 |
|  | % | -15.4 | -14.3 | -8.3 | -41.7 | -72.7 | -22.2 | -31.3 | -53.3 | -52.9 | -40.0 | -45.0 | +71.4 | -32.3 |
|  | Central America | 6 | 6 | 9 | 8 | 9 | 11 | 12 | 3 | 10 | 5 | 5 | 7 | 91 |
|  | % | +20.0 | +20.0 | +28.6 | +14.3 | +125.0 | +175.0 | +200.0 | -57.1 | +100.0 | -37.5 | -50.0 | +75.0 | +30.0 |
|  | East Asia | 38 | 38 | 34 | 28 | 32 | 32 | 35 | 41 | 21 | 33 | 39 | 46 | 417 |
|  | % | -7.3 | -19.1 | -26.1 | +16.7 | +3.2 | -30.4 | -23.9 | -2.4 | -63.2 | -17.5 | +50.0 | +27.8 | -13.5 |
|  | Japan | 19 | 16 | 16 | 8 | 12 | 11 | 13 | 21 | 8 | 15 | 16 | 15 | 170 |
|  | % | -17.4 | -38.5 | -40.7 | -42.9 | 0.0 | -50.0 | -31.6 | +50.0 | -63.6 | -6.3 | 0.0 | -16.7 | -25.8 |
|  | Europe | 88 | 91 | 84 | 82 | 65 | 78 | 72 | 84 | 65 | 72 | 74 | 72 | 927 |
|  | % | -10.2 | +5.8 | -1.2 | 7.9 | -15.6 | -29.1 | -33.9 | -10.6 | -35.0 | -39.5 | -20.4 | -10.0 | -17.7 |
|  | Middle East | 22 | 22 | 14 | 20 | 14 | 13 | 13 | 11 | 11 | 19 | 18 | 16 | 193 |
|  | % | +83.3 | +175.0 | -6.7 | +42.9 | -17.6 | -13.3 | +18.2 | -42.1 | -8.3 | -13.6 | +20.0 | +23.1 | +11.6 |
|  | North America | 302 | 290 | 311 | 269 | 230 | 236 | 251 | 325 | 315 | 318 | 273 | 301 | 3421 |
|  | % | +22.8 | +18.4 | +13.9 | -7.6 | -21.8 | -13.2 | -18.5 | +8.3 | +9.0 | +2.3 | +14.2 | +10.7 | +2.4 |
|  | Canada | 33 | 36 | 32 | 43 | 45 | 34 | 34 | 31 | 28 | 40 | 29 | 36 | 421 |
|  | % | +6.5 | +2.9 | -13.5 | +34.4 | +21.6 | -22.7 | -5.6 | -11.4 | -33.3 | -2.4 | -14.7 | +9.1 | -3.7 |
|  | Mexico | 12 | 11 | 13 | 7 | 10 | 10 | 6 | 10 | 9 | 20 | 11 | 8 | 127 |
|  | % | +20.0 | +37.5 | +116.7 | -36.4 | -9.1 | +25.0 | -53.8 | +25.0 | 0.0 | +100.0 | -21.4 | -46.7 | +3.3 |
|  | United States | 289 | 283 | 300 | 245 | 213 | 225 | 243 | 318 | 305 | 297 | 264 | 292 | 3274 |
|  | % | +22.5 | +22.0 | +13.6 | -13.4 | -24.7 | -12.5 | -15.3 | +12.4 | +16.4 | +0.3 | +18.9 | +15.9 | +3.7 |
|  | North Asia | 23 | 22 | 16 | 20 | 12 | 19 | 18 | 21 | 13 | 19 | 30 | 23 | 236 |
|  | % | +109.1 | 0.0 | -5.9 | 0.0 | -36.8 | -34.5 | -30.8 | -22.2 | -40.9 | +5.6 | +57.9 | +53.3 | -3.7 |
|  | Pacifica | 19 | 19 | 17 | 19 | 29 | 14 | 16 | 15 | 18 | 21 | 18 | 14 | 219 |
|  | % | -26.9 | +11.8 | -15.0 | +5.6 | +45.0 | -39.1 | -5.9 | -11.8 | +50.0 | +10.5 | +12.5 | -6.7 | -0.5 |
|  | South America | 21 | 20 | 15 | 12 | 12 | 11 | 17 | 13 | 15 | 15 | 16 | 17 | 184 |
|  | % | +40.0 | +42.9 | +36.4 | -29.4 | -25.0 | -26.7 | -22.7 | +116.7 | -16.7 | -21.1 | +60.0 | +30.8 | +4.5 |
|  | South Asia | 6 | 10 | 10 | 4 | 4 | 4 | 8 | 2 | 10 | 7 | 6 | 8 | 79 |
|  | % | 0.0 | +25.0 | +42.9 | -42.9 | -42.9 | -60.0 | 0.0 | -71.4 | +42.9 | -12.5 | +50.0 | -11.1 | -10.2 |
|  | Southeast Asia | 9 | 12 | 11 | 16 | 12 | 17 | 15 | 3 | 7 | 9 | 11 | 12 | 134 |
|  | % | -10.0 | +20.0 | -31.3 | +128.6 | +9.1 | +6.3 | -11.8 | -83.3 | 0.0 | -25.0 | +22.2 | -14.3 | -8.8 |
| * Percentage change compared the number of clinical trials for other than COVID-19 in 2020 with those in the same month of 2019. | | | | | | | | | | | | | | |

Titles and legends to supplementary figures

**Supplementary FIGURE 1** Percentage change of the number of newly started clinical trials for disease other than COVID-19 in each region, April 2020

Studies with no locations are not included in the counts or on the map. Studies with multiple locations are included in each region containing locations. The numbers show the percentage change compared the number of clinical trials for other than COVID-19 in 2020 with those in the same month of 2019.

**Supplementary FIGURE 2** Relationship between total cases of COVID-19 and percentage change of the number of newly started clinical trials for diseases other than COVID-19, April 2020

The size of the bubbles reflected the number of studies.

**Supplementary FIGURE 3** Relationship between total cases of COVID-19 and percentage change of the average monthly number of newly started clinical trials

(A) All regions; (B) regions with more than 30 studies. The size of the bubbles reflected the number of studies.

**Supplementary FIGURE 4** Percentage change of clinical trials with results in each region, May 2020

Studies with no locations are not included in the counts or on the map. Studies with multiple locations are included in each region containing locations. The numbers show the percentage change compared the number of clinical trials for other than COVID-19 in 2020 with those in the same month of 2019.

**Supplementary FIGURE 5** Relationship between total cases of COVID-19 and percentage change of the number of clinical trials with results, May 2020

The size of the bubbles reflected the number of studies.

**Supplementary FIGURE 6** Relationship between total cases of COVID-19 and percentage change of the average monthly number of clinical trials with results

(A) All regions; (B) regions with more than 30 studies. The size of the bubbles reflected the number of studies.

**Supplementary FIGURE 7** Number of completed clinical trial with results

(A) Number of completed clinical trials for diseases other than COVID-19 with results; (B) Percentage change of completed clinical trials for diseases other than COVID-19 with results.
