# Supplementary figures and images for: COVID-19 Pandemic and Trends in Clinical Trials: A Multi-Region and Global Perspective
Source: Front Med (Lausanne). 2021 Dec 24;8:812370. doi: 10.3389/fmed.2021.812370 (PMC8739772; doi:10.3389/fmed.2021.812370)

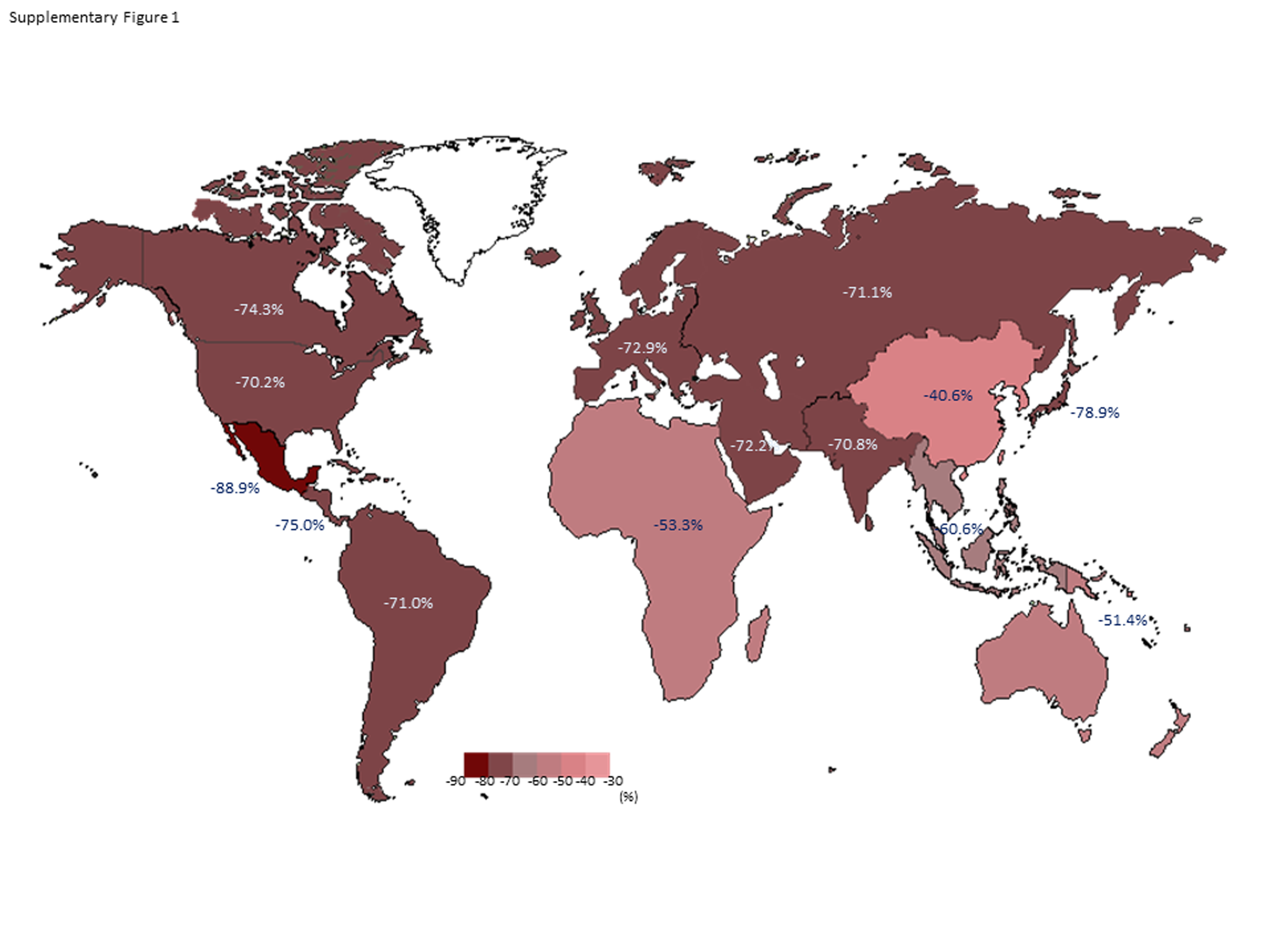

Supplement: Supplementary file 2 [file Image_1.TIF]

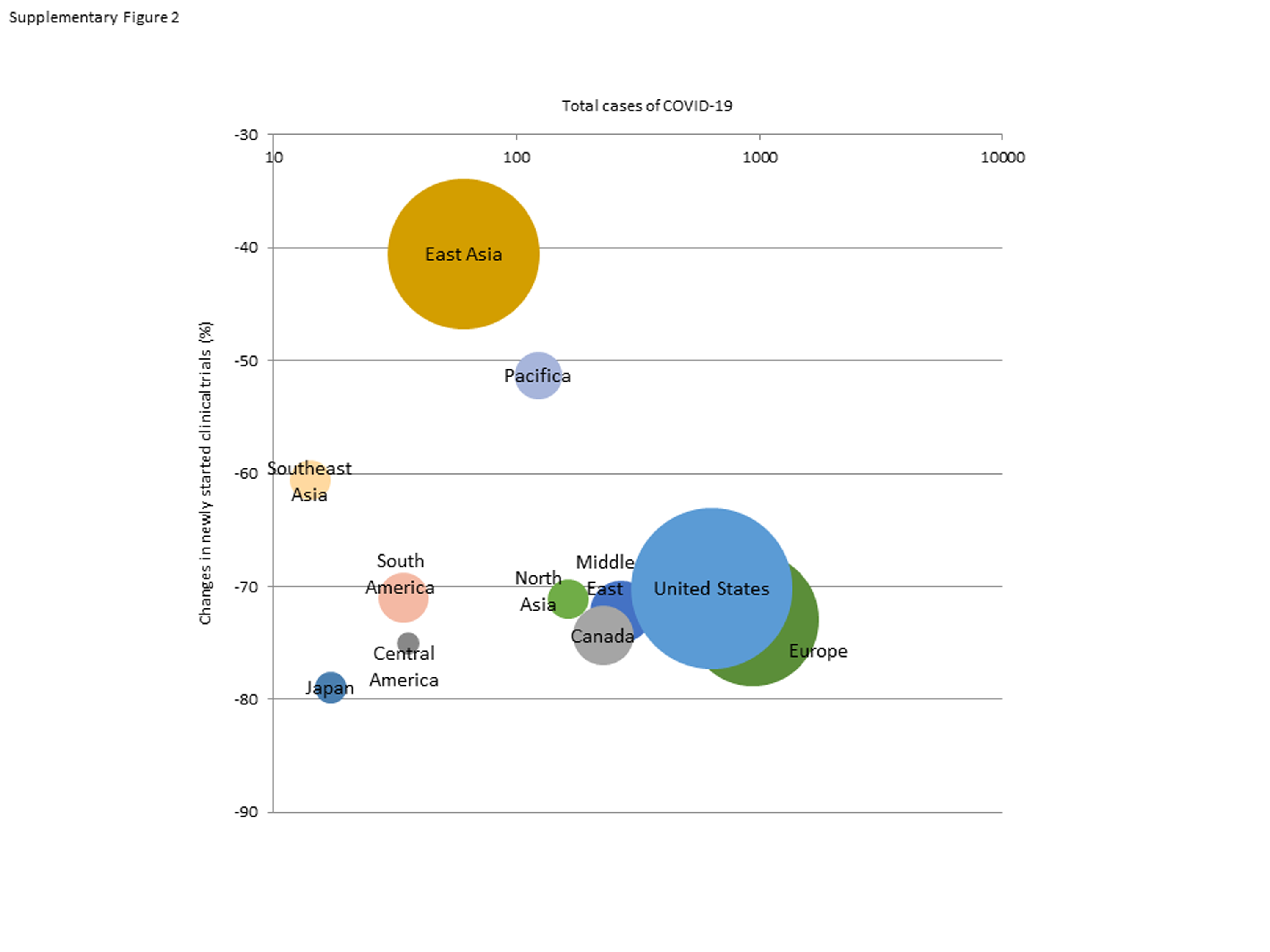

Supplement: Supplementary file 3 [file Image_2.TIF]

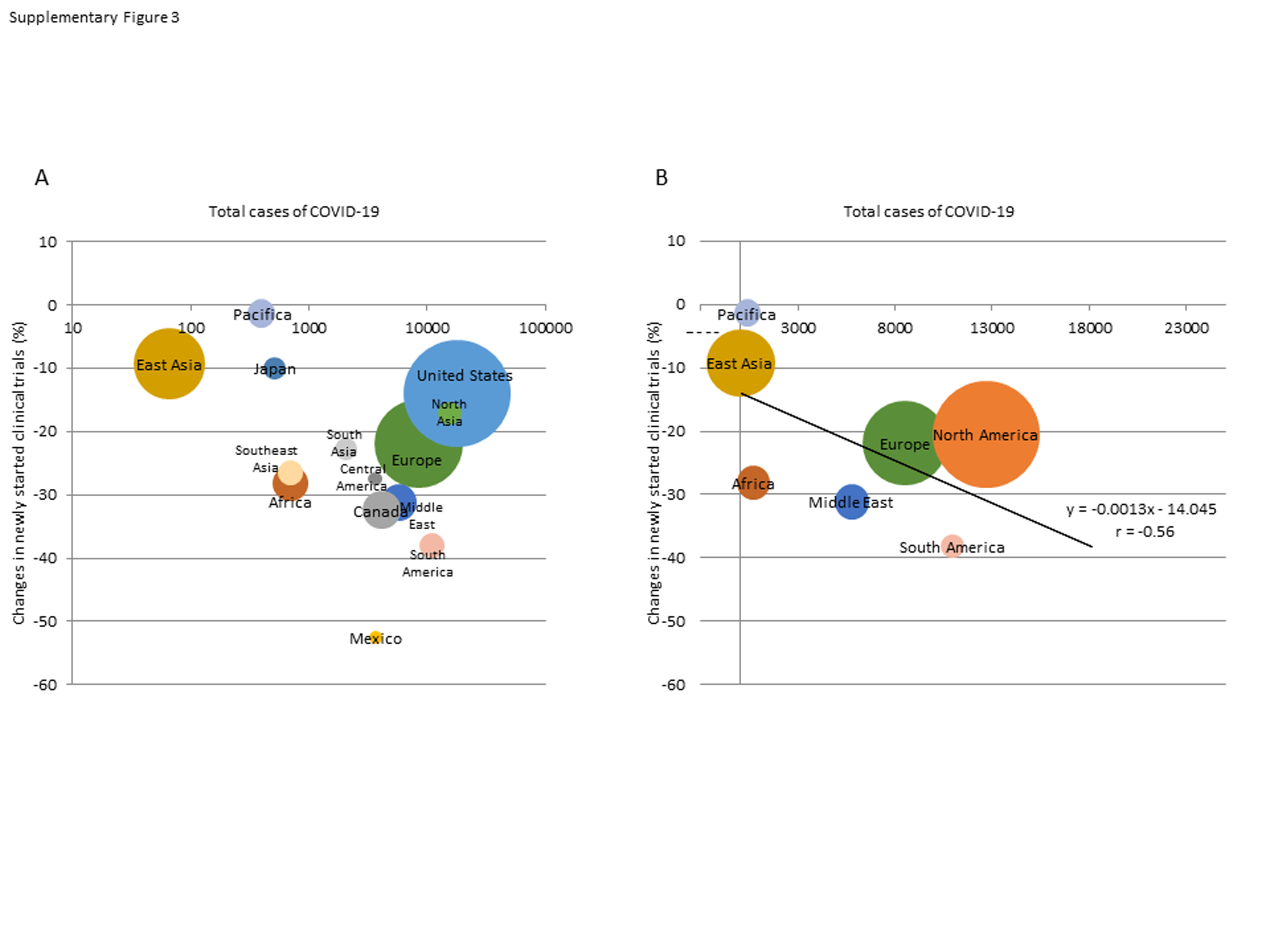

Supplement: Supplementary file 4 [file Image_3.TIF]

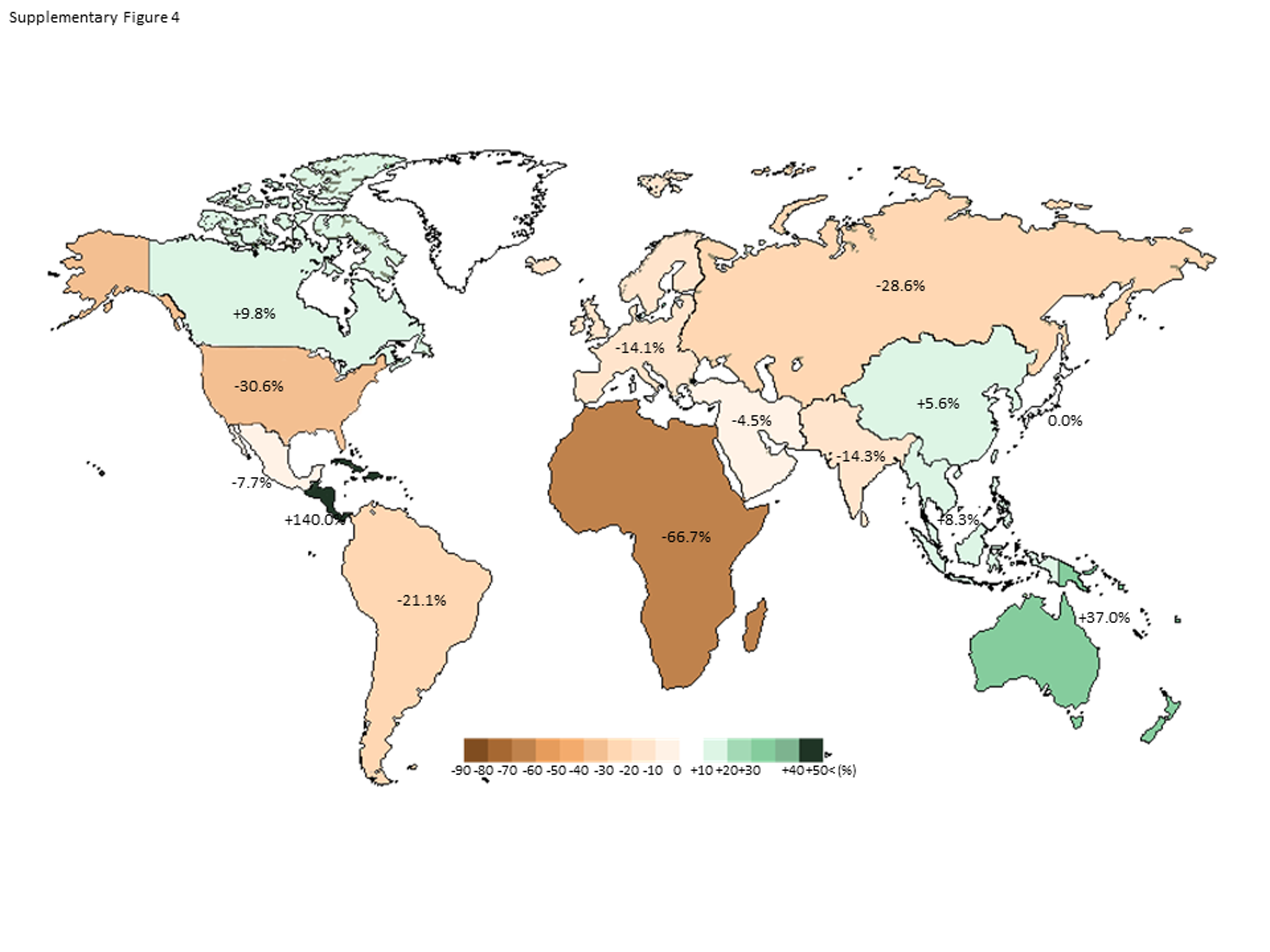

Supplement: Supplementary file 5 [file Image_4.TIF]

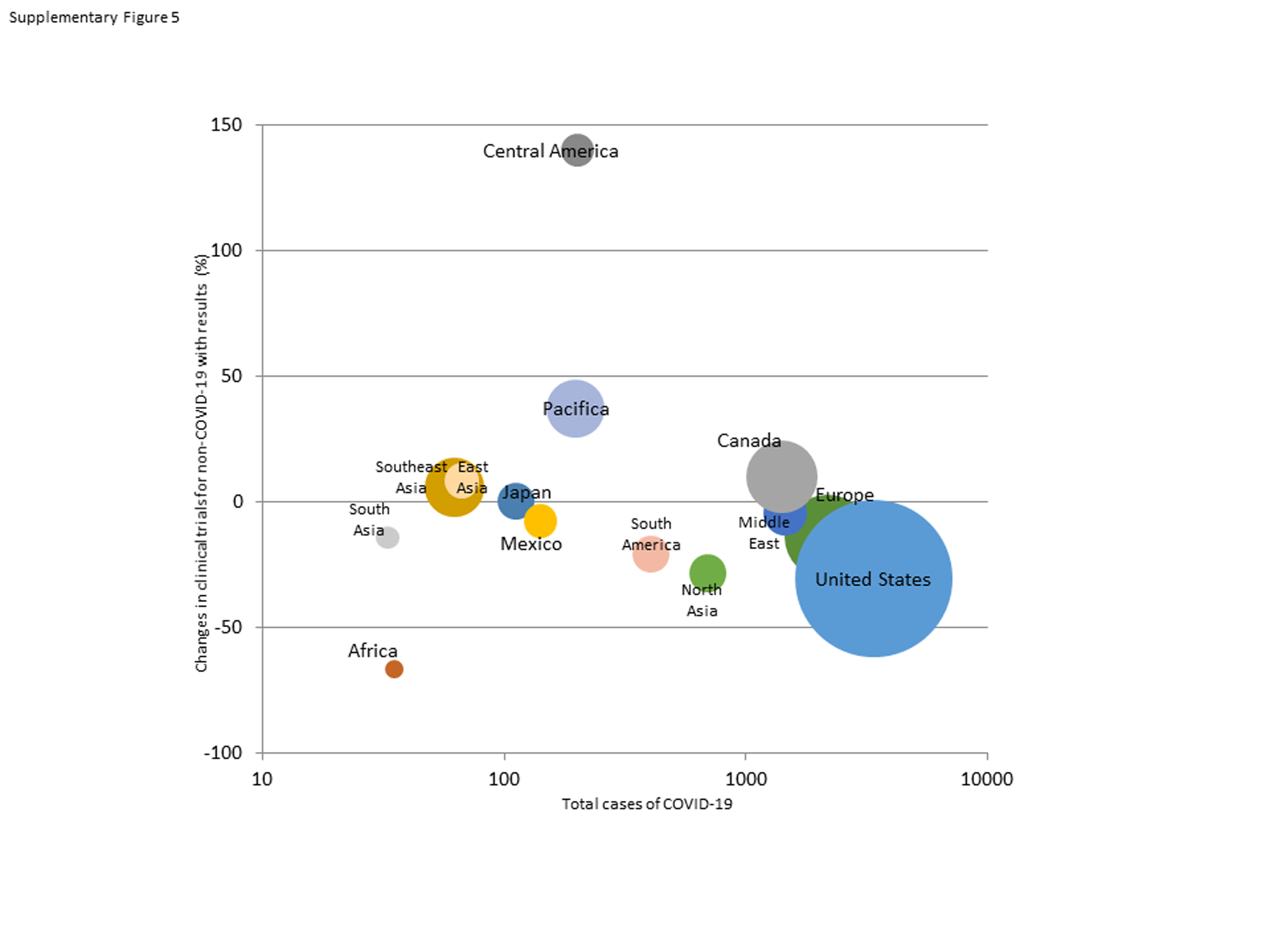

Supplement: Supplementary file 6 [file Image_5.TIF]

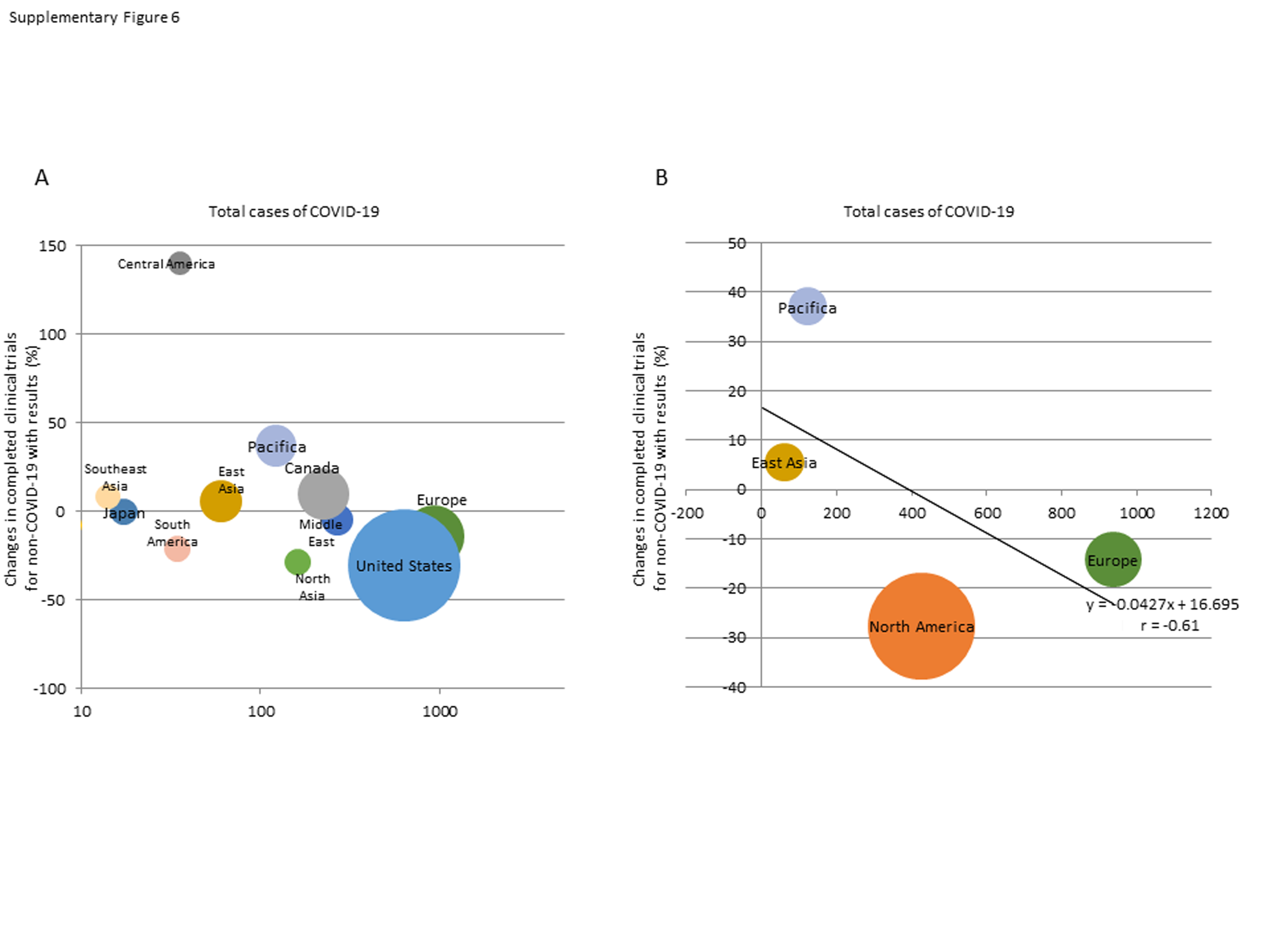

Supplement: Supplementary file 7 [file Image_6.TIF]

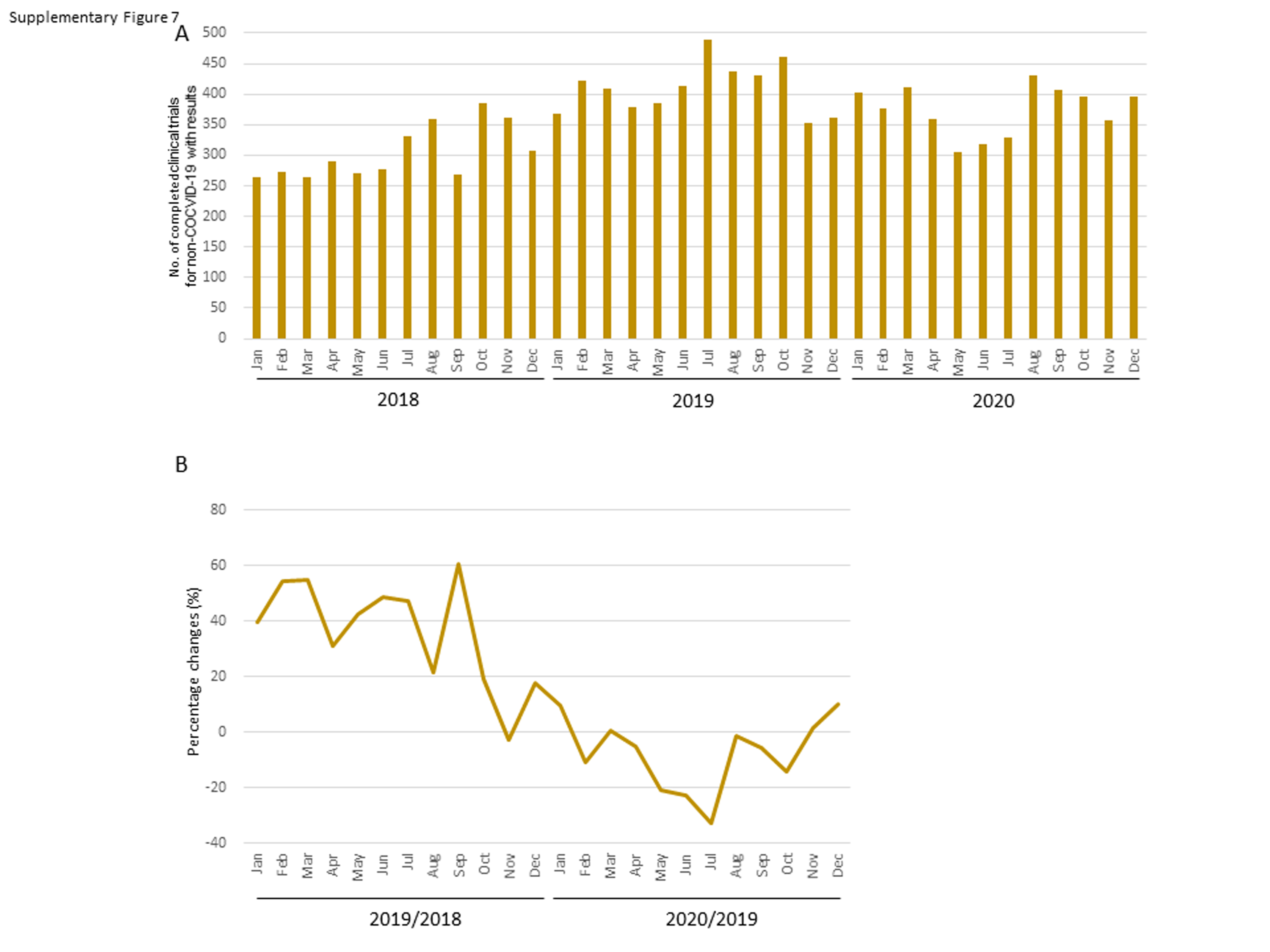

Supplement: Supplementary file 8 [file Image_7.TIF]
